# A New Representative of Star-Shaped Fungi: *Astraeus sirindhorniae* sp. nov. from Thailand

**DOI:** 10.1371/journal.pone.0071160

**Published:** 2014-05-07

**Authors:** Cherdchai Phosri, Roy Watling, Nuttika Suwannasai, Andrew Wilson, María P. Martín

**Affiliations:** 1 Department of Biology, Faculty of Science, Nakhon Phanom University, Nakhon Phanom, Thailand; 2 Caledonian Mycological Enterprises, Edinburgh, Scotland, United Kingdom; 3 Department of Biology, Faculty of Science, Srinakharinwirot University, Bangkok, Thailand; 4 Department of Botany and Plant Pathology, Purdue University, West Lafayette, Indiana, United States of America; 5 Departamento de Micología, Real Jardín Botánico, RJB-CSIC, Madrid, Spain; Cinvestav, Mexico

## Abstract

Phu Khieo Wildlife Sanctuary (PKWS) is a major hotspot of biological diversity in Thailand but its fungal diversity has not been thouroughly explored. A two-year macrofungal study of this remote locality has resulted in the recognition of a new species of a star-shaped gasteroid fungus in the genus *Astraeus*. This fungus has been identified based on a morphological approach and the molecular study of five loci (LSU nrDNA, 5.8S nrDNA, *RPB*1, *RPB2* and *EF1*-a). Multigene phylogenetic analysis of this new species places it basal relative to other *Astraeus*, providing additional evidence for the SE Asian orgin of the genus. The fungus is named in honour of Her Majesty Princess Sirindhorn on the occasion the 84th birthday of her father, who have both been supportive of natural heritage studies in Thailand.

## Introduction

Tropical rain forests are important terrestrial ecosystems. They harbour tremendous biodiversity and several of them are recongized as biodiversity hotspots [1]. Most of the attention paid to this biodiversity has focused on the fauna and flora at the expense of less charasmatic organisms such as fungi. In 1991, Hawksworth [2] has estimated that the number of fungi worldwide ultimately will be around 1.5 million species with fungal diversity considered to be highest in the tropical forests. More recently the estimated number of fungal species has been estimated anywhere between 3.5–5.1 million species [3]. According to Hibbett et al. [4] the overall rate of fungal species discovered worldwide has been fairly level for the last 10 years with a range of 1000–1200 new species reported per year, in both Basidiomycota and Ascomycota but mainly in the latter. Herein a new basidiomycete is added.

The current project is part of an effort to document the diversity of EM fungi associated with a broad range of host plants at a variety of spatial scales in Phu Khieo Wildlife Sanctuary (abbrev.: PKWS) of northeastern Thailand. This project was lead by a team of biologists from Nakhon Phanom University (NPU), in collaboration with Pibulsongkram Rajabhat University (PSRU), Srinakharinwirot University (SWU), Chulalongkorn University (CU), Real Jardín Botánico (RJB-CSIC, Madrid, Spain) and Caledonian Mycological Enterprises (Scotland, UK). The PKWS is a tropical region with a relatively high concentration of ectomycorrhizal associations. It is located in Chaiyaphum province, consisting of a complex of eigth contiguous protected areas in the western part of NE Thailand, and covers and area of 4,594 square kilometers. The western Isan forest complex is the only sizeable expanse of closed forest remaining in the region. It is unique in that it is able to sustain viable populations of wildlife species requiring large home ranges (e.g. tigers and elephants) [5]. It is also important in supporting a range of IUCN Red Listed animals and birds and is essential for conserving water resources in what is otherwise a hot and dry environment [6].

**Figure 1:**
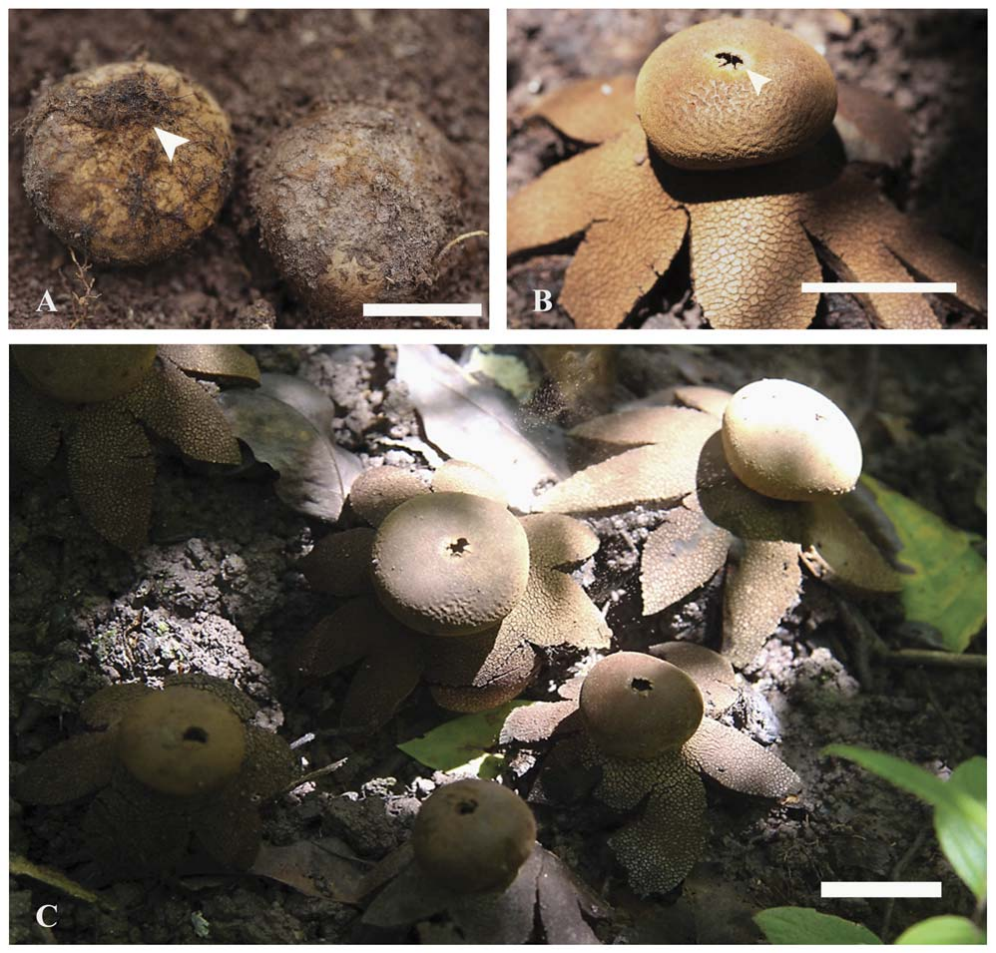
*Astraeus sirindhorniae* from the field. (A) immature basidiomes with basal rhizomorphs (arrowhead), bar = 17 mm. (B) mature basidiome split to form a series of rays revealing an endoperidium with an apical opening (arrowhead), bar = 24 mm. (C) basidiospores shooting from an opening apical (in blue circle), bar = 25 mm.

The scant research on fungi for the area has been partly addressed by excursions to describe the macrofungi associated with deciduous and mixed deciduous forest with pine. During the rainy season (July–September) in both 2010 and 2011, a subepigeous, gasteroid fungus was encountered exhibiting characteristics associated with the genus *Astraeus* Morgan (Order Boletales, clade Sclerodermatineae in [7]) and *Geastrum* Pers (Order Geastrales in [8]) (Fig. 1). The goal of this study is to identify the phylogenetic placement of this fungus using sequences of LSU nrDNA, 5.8S nrDNA, *RPB*1, *RPB2* and *EF1*-a, as well as compare its morphology to that of other species of star-shaped gasteroid fungi.

## Materials and Methods

### Fungal specimens

All necessary permits were obtained for the described field studies issued by Department of National park, wildlife & plant conservation, Bangkok, Thailand (Reference document number 0907.1/17723).

Basidiomes were collected in Phu Khieo Wildlife Sanctuary, Chaiyaphum province, Thailand, during the months of July and September, 2010 and 2011. Field characters such as peridial and glebal colours (Colour identification chart, Royal Botanic Garden, E, 1969) and textures, etc. were recorded in the field and in the laboratory. Basidiospores were mounted in Melzer's reagent [9] and examined and photographed using light microscopy at magnifications of 400–1000× (DIC BX51 Olympus). Mean spore size and range was determined by measuring the diameter of at least 30 spores. Ornamentations were described and later analysed using scanning electron microscopy (SEM). For SEM, spore samples were air-dried, mounted, and sputter-coated with gold before being scanned using a JEOL JSM-840 scanning electron microscope. Peridium structure was examined under polarization microscopy (Imager A1, Zeiss). Attempts to culture the mycelium from fresh basidiomes using a modified Melin Norkrans's medium (MMN) were unsuccessful. Specimens are deposited in BBH, E and MA-Fungi.

### DNA isolation, amplification and sequencing

Genomic DNA was extracted from specimens mentioned in Table 1. DNeasy Plant Mini Kit (Qiagen) was used according to the manufacturer's instructions. Five loci were amplified: a) the partial of 5′ end of nuclear ribosomal large subunit RNA gene sequences (nrLSU) with primers LR0R, LR3R, LR5, and LR7 [10]; b) the internal transcribed spacer of nuclear ribosomal DNA (ITS) with primers ITS1F and ITS4B [11]; c) the largest subunit of RNA polymerase II gene sequences (*RPB*1) with primers RPB1-Af (5′-GAR TGY CCD GGD CAY TTY GG-3′) and RPB1-Cr (5′-CC NGC DAT NTC RTT RTC CAT RTA-3′) [12]; d) the second largest subunit of RNA polymerase II gene sequences (*RPB*2) with primers RPB2-f5F (5′-GAY GAY MGW GAT CAY TTY GG-3′) [13] and RPB2-b7R (5′-GAY TGR TTR TGR TCR GGG AAV GG-3′) [14]; and e) the transcription elongation factor 1-alpha (*EF1*-a) with primers 983F (5′-GCY CCY GGH CAY CGT GAY TTY AT-3′) and 2218R (5′-ATG ACA CCR ACR GCR ACR GTY TG-3′) [15]. Polymerase chain reactions (PCR) contained 0.4 U Phire Hot Start II DNA Polymerase (Finnzymes, Sweden), 1× Phire Plant PCR Buffer with 1.5 mM MgCl_2_, 200 µM of each dNTP and 0.5 µM of each primer. The ITS amplification was run on an Eppendorf thermocycler (Eppendorf, Germany) using the following parameters: initial denaturation of 5 min at 98°C, followed by 40 cycles each with a denaturation step of 5 s at 98°C, annealing for 5 s at 57°C, an elongation step of 20 s at 72°C, and a final elongation step of 10 min at 72°C. The same conditions were used for nrLSU, *RPB*1, *RPB*2 and *EF1*-a amplification except that the annealing temperatures were 50°C, 55°C, 55°C and 57°C, respectively. Amplicons were purified using the QIAquick PCR Purification Kit (Qiagen) and then sequenced at the 1^st^ BASE laboratories Sdn Bhd (Malaysia). Except for *RPB*2 amplicon was cloned using TA cloning kit (Invitrogen) into *Escherichia coli* TOP10 before sequenced. Sequences were assembled and edited with BioEdit [16]. BLASTN queries with MEGABLAST option were used to compare sequences obtained against sequences in the National Center of Biotechnology Information (NCBI) nucleotide database [17]. All new sequences have been deposited on the EMBL-EBI database and their accession numbers are presented in Table 1.

**Table 1:** List of specimens in this study.

Genus	Species	ID/Herbarium ID	Location/Citation	ITS	nrLSU	RPBI	RPBII	EF1-a
***Astraeus***	***sirindhorniae***	GAPK1/E30288	PKWS, Chaiyaphum	HE681772	HE68182	HE68191	KC854536	KC854542
***Astraeus***	***sirindhorniae***	GAPK2/MA-Fungi82080	PKWS, Chaiyaphum	HE681773	HE68183	HE68192	KC854538	KC854543
***Astraeus***	***sirindhorniae***	GAPK3/BBH34831	Chaing Mai	HE681774	HE68184	HE68193	KC854539	KC854544
***Astraeus***	***sirindhorniae***	GAPK4/BBH34830	PKWS, Chaiyaphum	HE681775	HE68185	HE68194	KC854541	KC854545
*Astraeus*	*asiaticus*	Arora 02-121	Thailand	EU718089	DQ644199	FJ536588	FJ536625	FJ536665
*Astraeus*	*asiaticus*	ASTRAE-44	Sri Lanka	AJ629395				
*Astraeus*	*asiaticus*	ASTRAE-56	Thailand	AJ629396				
*Astraeus*	*asiaticus*	ASTRAE-64	Thailand	AJ629400				
*Astraeus*	*asiaticus*	ASTRAE-65	Thailand	AJ629401				
*Astraeus*	*hygrometricus*	Bneil (MB 05-029)	Massachusetts USA	EU718087	DQ682996	FJ536586	FJ536623	FJ536663
*Astraeus*	*hygrometricus*	AWW220^a^	Massachusetts USA	FJ710187				
*Astraeus*	*hygrometricus*	ASTRAE-73^a^	Wisconsin, USA	AJ629398				
*Astraeus*	*hygrometricus*	ASTRAE-86^a^	Michigan, USA	AJ629403				
*Astraeus*	*hygrometricus*	ASTRAE-87^b^	Greece	AJ629404				
*Astraeus*	*hygrometricus*	ASTRAE-72^b^	Spain	AJ629408				
*Astraeus*	*hygrometricus*	ASTRAE-74^a^	Wisconsin, USA	AJ629399				
*Astraeus*	*hygrometricus*	ASTRAE-43	France	AJ629406				
*Astraeus*	*hygrometricus*	ASTRAE-42	France	AJ629394				
*Astraeus*	*odoratus*	ASTRAE-61	Thailand	AJ6298776				
*Astraeus*	*odoratus*	ASTRAE-62	Thailand	AJ629877				
*Astraeus*	*pteridis*	Ashy 3	Switzerland	EU718088	AF336238	FJ536587	FJ536624	FJ536664
*Astraeus*	*pteridis*	PDD88503	New Zealand	FJ710188	EU718158			
*Astraeus*	*pteridis*	ASTRAE-36	Mexico	AJ629392				
*Astraeus*	*pteridis*	ASTRAE-25	Wisconsin, USA	AJ629410				
*Astraeus*	*pteridis*	ASTRAE-24	Wisconsin, USA	AJ629409				
*Astraeus*	*pteridis*	ASTRAE-37	Spain	AJ629393				
*Boletinellus*	*merulioides*	MB 02-199	Massachusetts USA	DQ200922	AY684153	DQ435803	DQ366281	DQ056287
*Boletinellus*	*merulioides*				AF336239			
*Boletinellus*	*merulioides*				AY612807			
*Boletinellus*	*rompelii*	No1192			EU718159			
*Calostoma*	*berkeleyi*	AWW268	Malaysia	EU718090	EU718128	FJ536589	FJ536626	FJ536666
*Calostoma*	*cinnabarinum*	AWW136	Massachusetts USA	AY854064	AY645054	AY780939	AY857979	AY879117
*Calostoma*	*Fuscum*	OKM 23918	Western Australia	EU718091	EU718129	FJ536590	FJ536627	
*Calostoma*	*Fuscum*	PDD70777		FJ710190	EU718161			
*Calostoma*	*insignis*	Arora 98-31	Thailand	EU718092	EU718130		FJ536628	
*Calostoma*	*japonicum*	TKG-SC-40701	Japan	EU718093	EU718131	FJ536591	FJ536629	
*Calostoma*	*junghuhnii*	VC1151	India		EU718163			
*Calostoma*	*lutescens*	1329		FJ710192	EU718164			
*Calostoma*	*orirubra*	HKAS32119	China	FJ710195	EU718165			
*Calostoma*	*rodwayi*	GMM 7572	New Zealand	EU718095	EU718133		FJ536631	
*Calostoma*	*sarasinii*	DED7660	Malaysia	EU718096	EU718134	FJ536593	FJ536632	FJ536668
*Calostoma*	Sp	HKAS38133	China	EU718097	EU718135		FJ536633	
*Calostoma*	Sp	HKAS38139	China	EU718098	EU718136	FJ536594	FJ536634	
*Diplocystis*	wrightii	DH2002			DQ534665			
*Gyroporus*	*aff. castaneus*	E4600			EU718169			
*Gyroporus*	*aff. castaneus*	E843c			EU718170			
*Gyroporus*	*aff. castaneus*	E4879c			FJ710208			
*Gyroporus*	*castaneus*	Gc1	Germany	EU718099	AF336252	FJ536595	FJ536635	FJ536669
*Gyroporus*	*castaneus*	239-97	USA	EU718100	AF336253	FJ536596	FJ536636	FJ536670
*Gyroporus*	*castaneus*	REH8804	Thailand	EU718101	EU718137	FJ536597	FJ536637	FJ536671
*Gyroporus*	*aff. cyanescens*	REH8821	Western Australia	EU718103	EU718139	FJ536599	FJ536639	FJ536673
*Gyroporus*	*aff. cyanescens*	E486	Australia		EU718173			
*Gyroporus*	*cyanescens*	MB 05-001	USA	EU718102	EU718138	FJ536598	FJ536638	FJ536672
*Gyroporus*	*cyanescens*	Gcy2	Germany		AF336254			
*Gyroporus*	*cyanescens*	E8758c	Australia		EU718171			
*Gyroporus*	*aff. cyanescens*	OKM23719	Western Australia	EU718104	EU718140	FJ536600	FJ536640	
*Gyroporus*	*purpurinus*	PRL 3737	Illinois, USA	EU718105	EU718141	FJ536601	FJ536641	FJ536674
*Gyroporus*	sp.	REH8799	Thailand	EU718106	EU718142	FJ536602	FJ536642	FJ536675
*Gyroporus*	sp.	Arora 00-429	Zimbabwe	EU718107	EU718143	FJ536603	FJ536643	FJ536676
*Gyroporus*	sp.	E8155			EF561627			
*Gyroporus*	sp.	REH8805			EU718175			
*Gyroporus*	*subalbellus*	OKM25477	Texas, USA	EU718108	EU718144	FJ536604	FJ536644	FJ536677
*Phlebopus*	*beniensis*	Omon 98.015			AY612822			
*Phlebopus*	*marginatus*	REH8883	Eastern Australia	EU718109	EU718145	FJ536605	FJ536645	FJ536678
*Phlebopus*	*marginatus*	MEL2145841	Australia		FJ600322			
*Phlebopus*	*portentosus*	php1	Africa	EU718110	AF336260	FJ536606	FJ536646	FJ536679
*Phlebopus*	sp.				AY612816			
*Phlebopus*	sp.	REH8795	Thailand	EU718111	AF336260	FJ536607	FJ536647	FJ536680
*Phlebopus*	*sudanicus*				AF336261			
*Pisolithus*	*albus*	PERTH4681	Australia	FJ710202	EU718176			
*Pisolithus*	*arhizus*				AF336262			
*Pisolithus*	*aurantioscabrosus*	AWW297	Malaysia	EU718112	EU718146	FJ536608	FJ536648	FJ536681
*Pisolithus*	sp.	ECV3205	California USA	EU718113	EU718147	FJ536609	FJ536649	
*Pisolithus*	*tinctorius*	AWW219	Massachusetts USA	EU718114	EU718148	FJ536610	FJ536650	FJ536682
*Scleroderma*	*areolatum*	AWW211	Massachusetts USA	EU718115	EU718149	FJ536611	FJ536651	FJ536683
*Scleroderma*	*areolatum*	PBM2208	W. Australia	EU718116	EU718150	FJ536612	FJ536652	FJ536684
*Scleroderma*	*bermudense*	BZ3961	Belize	EU718118	DQ644137	FJ536614	FJ536654	FJ536686
*Scleroderma*	*citrinum*	AWW212	Massachusetts USA	EU718119	EU718151	FJ536615	FJ536655	FJ536687
*Scleroderma*	*citrinum*				AF336266			
*Scleroderma*	*columnare*				AF261533			
*Scleroderma*	*columnare*				AF336273			
*Scleroderma*	*echinatum*				AF336268			
*Scleroderma*	*fuscum*	Trappe26575			EU718178			
*Scleroderma*	*leave*	MCA242	North Carolina USA	EU718117	DQ677138	FJ536613	FJ536653	FJ536685
*Scleroderma*	*leave*	OSC27936		EU718120	DQ683003	FJ536616		
*Scleroderma*	*mcalpinei*	OSC 24605		EU718122	DQ682999		FJ536657	
*Scleroderma*	*meridionale*	AWW218	Massachusetts USA	EU718121	EU718152	FJ536617	FJ536656	FJ536688
*Scleroderma*	*polyrhizum*	AWW216	Massachusetts USA	EU718123	EU718153	FJ536618	FJ536658	FJ536689
*Scleroderma*	*sinnamariense*	AWW254	Malaysia	EU718124	EU718154	FJ536619	FJ536659	FJ536690
*Scleroderma*	sp.	HKAS43607			FJ710210			
*Scleroderma*	sp.	Arora9917			EU718179			
*Scleroderma*	sp.	MCA2168			EU718180			
*Scleroderma*	sp.	MEL2295738			EU718181			
*Scleroderma*	sp. Brown	AWW311	Malaysia	EU718126	EU718156	FJ536621	FJ536661	FJ536692
*Scleroderma*	sp. White	AWW260	Malaysia	EU718125	EU718155	FJ536620	FJ536660	FJ536691
*Scleroderma*	*verrucosum*				AF336271			
*Tremellogaster*	*surinamensis*	MCA 1985	Guyana	EU718127	DQ534664	FJ536622	FJ536662	FJ536693

Notes:

a New species described in Phosri et al. [32]: *A. smithii* and ASTRAE-86 is the holotype.

b New species described in Phosri et al. [32]: *A. telleriae* and ASTRAE-87 is the holotype.

**Figure 2:**
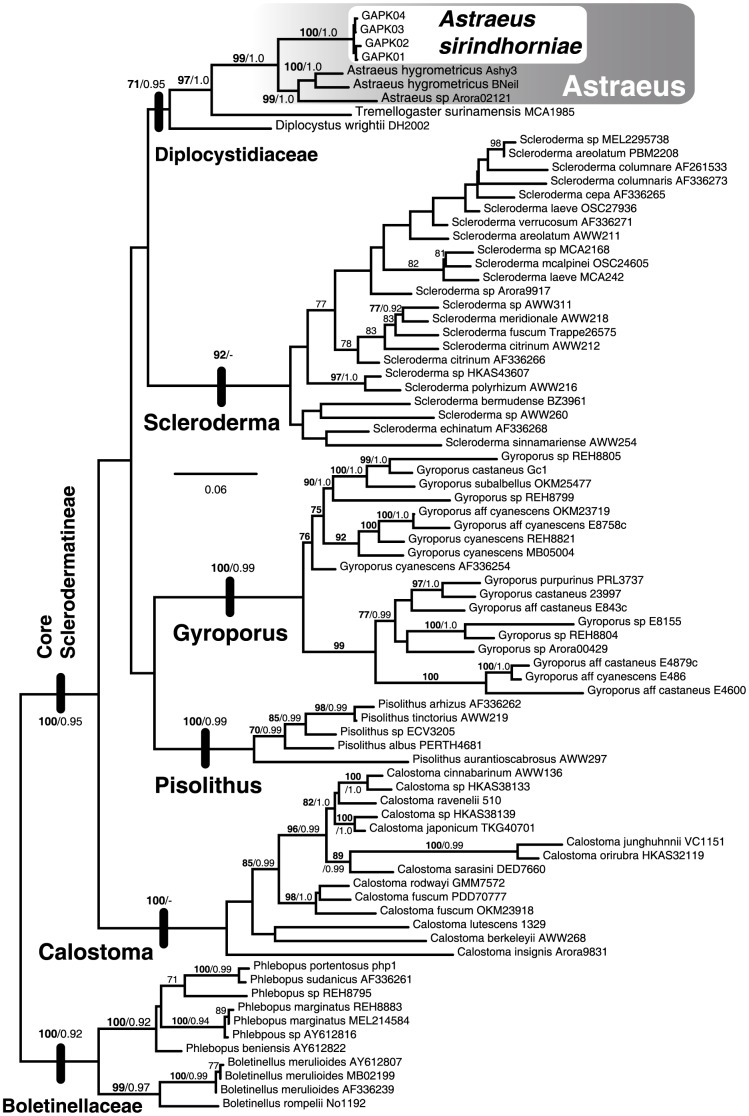
Maximum likelihood tree from a multigene dataset reveals the placement of *Astraeus sirindhorniae* within the Sclerodermatineae. Thick vertical black bars identify root branch for the taxonomic lineage indicated by the adjacent label. Numbers above branches identify the statistics bootstrap percentages (bold text, before forward slash) and Bayesian posterior probabilities (normal text, after forward slash) for that branch. Maximum likelihood bootstraps from 1000 iterations. Bayesian posterior probabilities from 1000 iterations (1 million runs sampling every 1000^th^ iteration).

**Figure 3:**
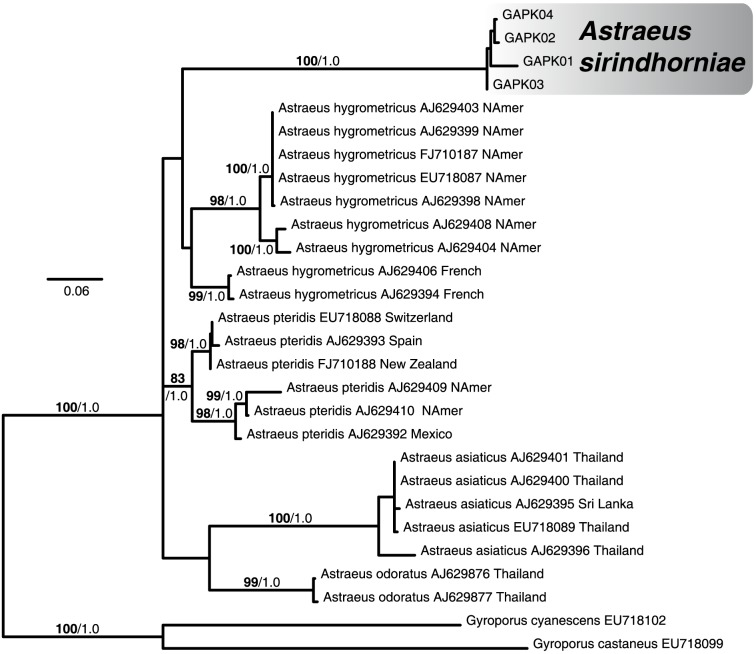
Maximum likelihood tree from ITS dataset identifies *Astraeus sirindhorniae* as a distinct species of *Astraeus*. Numbers above branches identify the statistics bootstrap percentages (bold text, before forward slash) and Bayesian posterior probabilities (normal text, after forward slash) for that branch. Maximum likelihood bootstraps from 1000 iterations. Bayesian posterior probabilities from 1000 iterations (1 million runs sampling every 1000^th^ iteration).

Specimen codes as indicated in Figure 2 and 3. All specimens from Phu Khieo Wildlife Sanctuary abbreviated as PKWS.

### Phylogenetic analysis

Two datasets were created for this study. One is a multigene dataset that examines the phylogenetic position of the gasteroid fungus from PKWS using ribosomal RNA and protein coding genes (nrLSU, the 5.8S region of the ITS, *RPB*1, *RPB*2 and *EF1*-a). Genes missing for individual samples were coded as “?” in the dataset to represent missing data. A second dataset consisted of only ITS sequence data to compare this taxon against other known *Astraeus* species. Both datasets, consisting of original sequences, plus sequences acquired from Genbank, were aligned using MUSCLE [18] with additional manual adjustments to the alignment performed in Mesquite 2.74 [19].

For each dataset, maximum likelihood and Bayesian analyses were performed using the CIPRES web portal (http://www.phylo.org/portal2/) [20]. Maximum likelihood bootstrapping analyses was performed on each dataset with RAxML 7.2.8 [21], using the default parameters as implemented on the CIPRES NSF XSEDE resource with bootstrap statistics calculated from 1000 bootstrap replicates. Bayesian phylogenetic analyses were performed using Mr Bayes v. 3.2.1 [22] on CIPRES XSEDE resource with default parameters (Nst = 6, with 2 runs, 4 chains per run, each run searching for 1000000 generations sampling every 1000^th^ generation).

### Nomenclature

The electronic version of this article in Portable Document Format (PDF) in a work with an ISSN or ISBN will represent a published work according to the International Code of Nomenclature for algae, fungi, and plants, and hence the new names contained in the electronic publication of a PLOS ONE article are effectively published under that Code from the electronic edition alone, so there is no longer any need to provide printed copies.

The new taxon described herein has been submitted to MycoBank and the unique MycoBank number provided can be used to retrieve the associated taxonomic information at http://www.mycobank.org/MycoTaxo.aspx?Link=T&Rec=.

## Results

### Phylogenetic analysis

BLAST searches with megablast option were used to compare the sequences obtained (nrLSU and ***RPB***1 around 1460 and 1310 bp, respectively) against the sequences in the National Center of Biotechnology Information (NCBI) nucleotide databases [17]. Sequences from the gasteroid fungus from PKWS produce matches for *Astraeus* spp., *Diplocystis wrightii* Berk. & M.A. Curtis, *Pisolithus* spp., *Scleroderma* spp., *Tremellogaster surinamensis* E. Fisch. and *Veligaster columnaris* (Berk. & Broome) Guzman. All of these taxa are gasteroid Boletales included in Sclerodermatineae [7].

To evaluate the phylogenetic position of the sclerodermatoid fungus from PKWS, a multigene dataset was created using nrLSU, 5.8S, *RPB*1, *RPB*2 and *EF1*-a genes from 80 specimens. This dataset was rooted using the Boletinellaceae (*Boletellus* and *Phlebopus*) while the genera *Astraeus*, *Calostoma*, *Diplocystis*, *Gyroporus*, *Phlebopus*, *Scleroderma* and *Tremellogaster* consisted of the ingroup. Maximum likelihood bootstrap (MLB) and Bayesian posterior probabilities (PP) strongly support a monophyletic placement for the sclerodermatoid fungus with *Astraeus* (MLB = 99%, PP = 1.0; Fig. 2). With it's inclusion in *Astraeus*, there is a strong sister relationship with the monotypic genus *Tremellogaster* (MLB = 97%, PP = 1.0) and weak support for the inclusion of these taxa, along with *Diplocystus* to form the Diplocystidiaceae (MLB = 71%, PP = 0.95). Sequences use for both phylogenetic datasets and their corresponding GenBank accession numbers are given in Table 1.

An ITS dataset was developed to evaluate the uniqueness of this new taxon relative to other *Astraeus* species. This dataset consists of 28 samples (2 outgroup samples from *Gyroporus*). Maximum likelihood and Bayesian phylogenetic analysis identifies eight major clades that can be recognized as species (each with MLB>98% and PP = 1.0; Fig. 3). Five of these represent taxa already defined by Phosri et al. [23]. Four samples of the new *Astraeus* taxon form a strongly supported group distinct from the other major *Astraeus* clades (MLB = 100%, PP = 1.0) which we will from now on refer to as *Astraeus sirindhorniae*.

### Taxonomy

*Astraeus sirindhorniae* sp. nov. Watling, Phosri, Sihanonth, A.W.Wilson & M.P. Martín

#### Mycobank

MB803956

#### Etymology

The species is named after Princess Sirindhorn on the occasion the 84th birthday of her father, who have both been supportive of natural heritage studies in Thailand and as a token of respect and recognition of the great interest shown by Her Majesty in the natural history and conservation of natural resources of Thailand. Now her name will be known in association with the Greek Titan of Astrology (*Astraeus*).

#### Holotype

Thailand, Phu Khieo Wildlife Sanctuary, Chaiyaphum, coll. C.Phosri, 9 September 2010, (BBH34830)

### Diagnostic description

#### Basidiomycota: Boletales: Sclerodermatineae

Large, subglobose to ellipsoid, subepigeous, dry basidiomes splitting at maturity to form a non-gelatinised, exoperidium with rays that unfold into a star-shaped structure. Enclosed within the exoperidium is a pale, thin, dry, stipitate endoperidium containing a powdery gleba of date-brown to umber (Colour identification chart, Royal Botanic Garden, E, 1969), large, globose, distinctly but minutely verrucose spores <11 µm diam. and lacking a columella.

**Figure 4:**
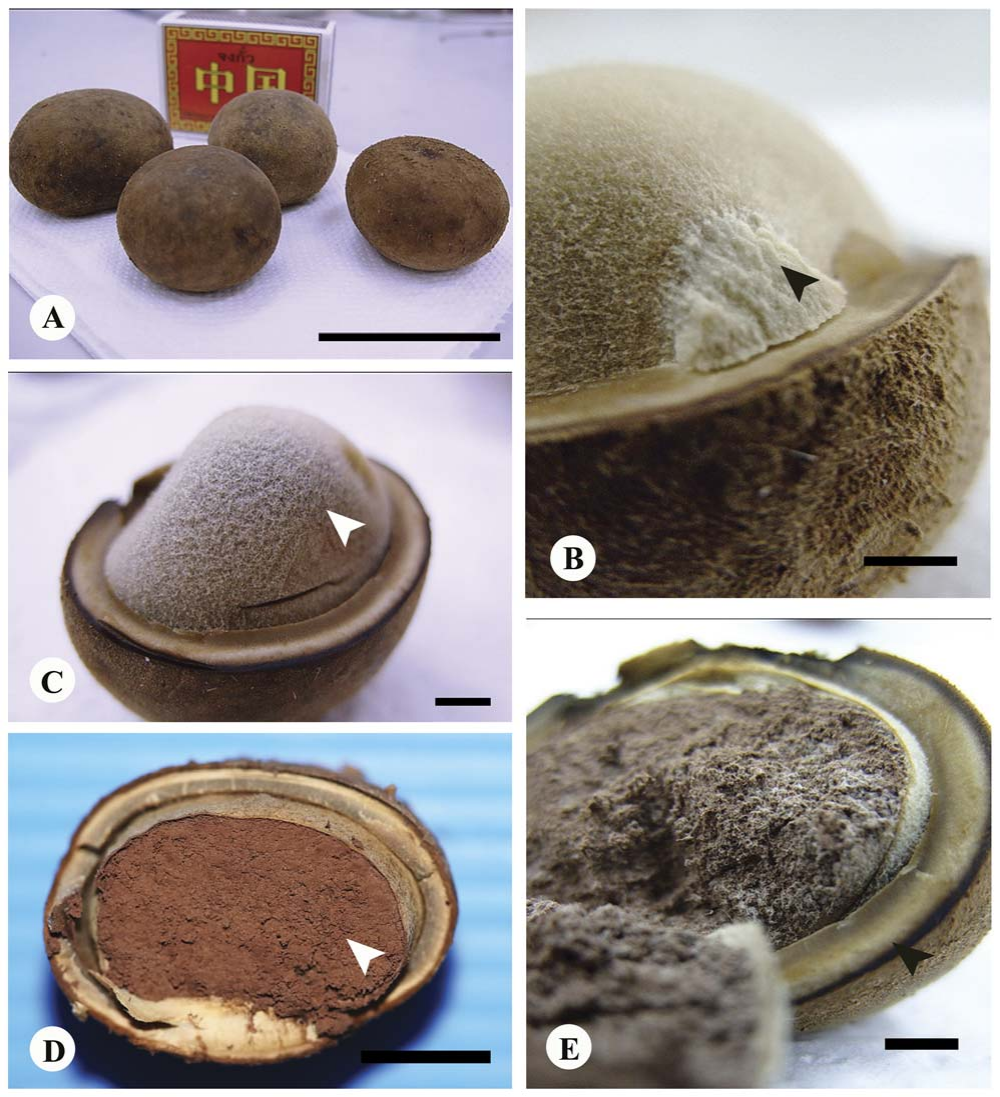
*Astraeus sirindhorniae*. (A) immature basidiomes, bar = 60 mm. (B) short stipitate endoperidium (arrowhead), bar = 3 mm. (C) fibrillose endoperidium (arrowhead), bar = 3 mm. (D) gleba colour become umber to date- brown when mature (arrowhead), bar = 10 mm. (E) complex outer peridium, bar = 3 mm.

**Figure 5:**
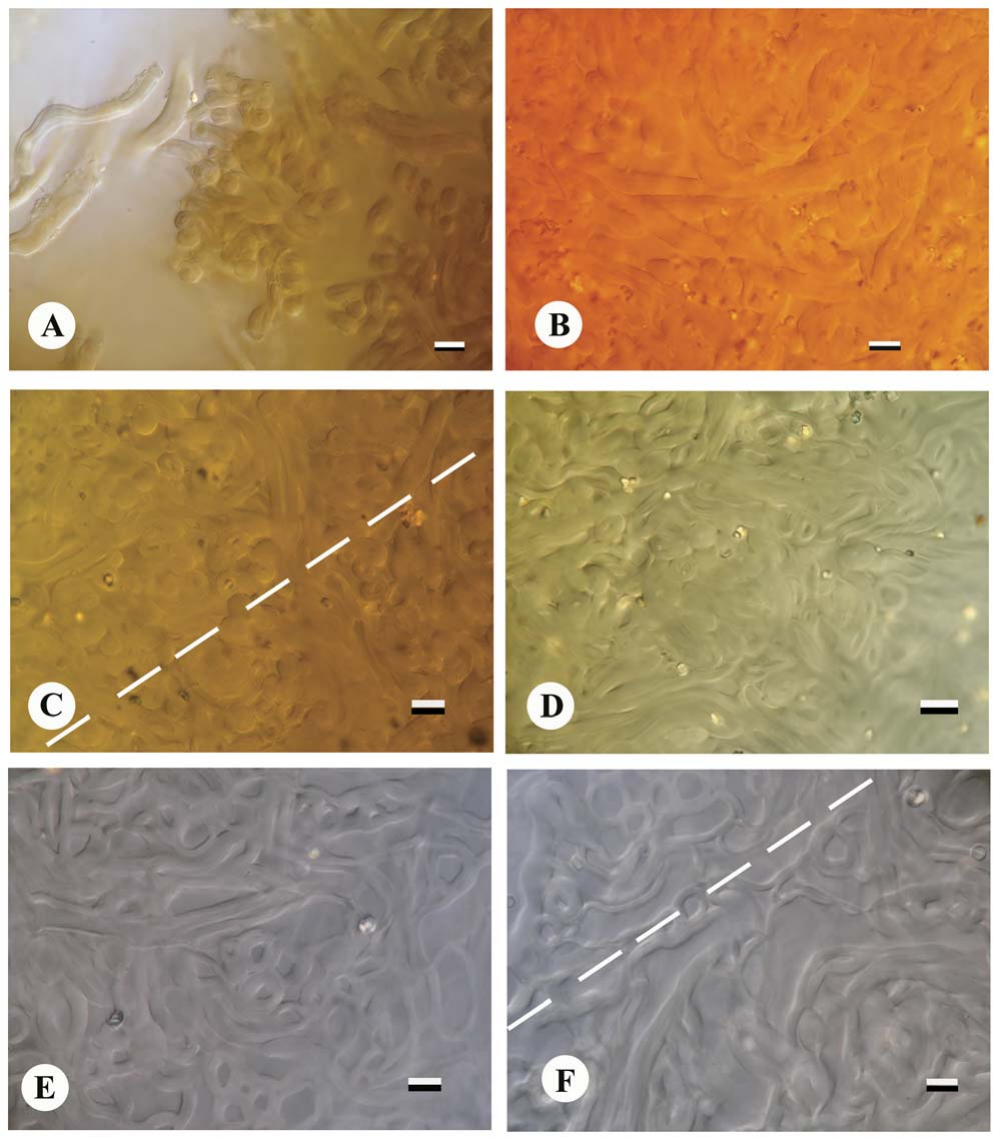
*Astraeus sirindhorniae*. Exoperidium layers. (A) exoperidial suprapellis, outer most surface, bar = 6 µm. (B) exoperidial suprapellis, bar = 7 µm. (C) interface layer between exoperidial suprapellis (top left) and mediopellis (lower right), bar = 6 µm. (D) exoperidial mediopellis, bar = 7 µm. (E) exoperidial mediopellis (inner most), bar = 7 µm. and (F) interface layers between exoperidial mediopellis (top left) and subpellis (lower right), bar = 8 µm. Magnification at 1,000×.

**Figure 6:**
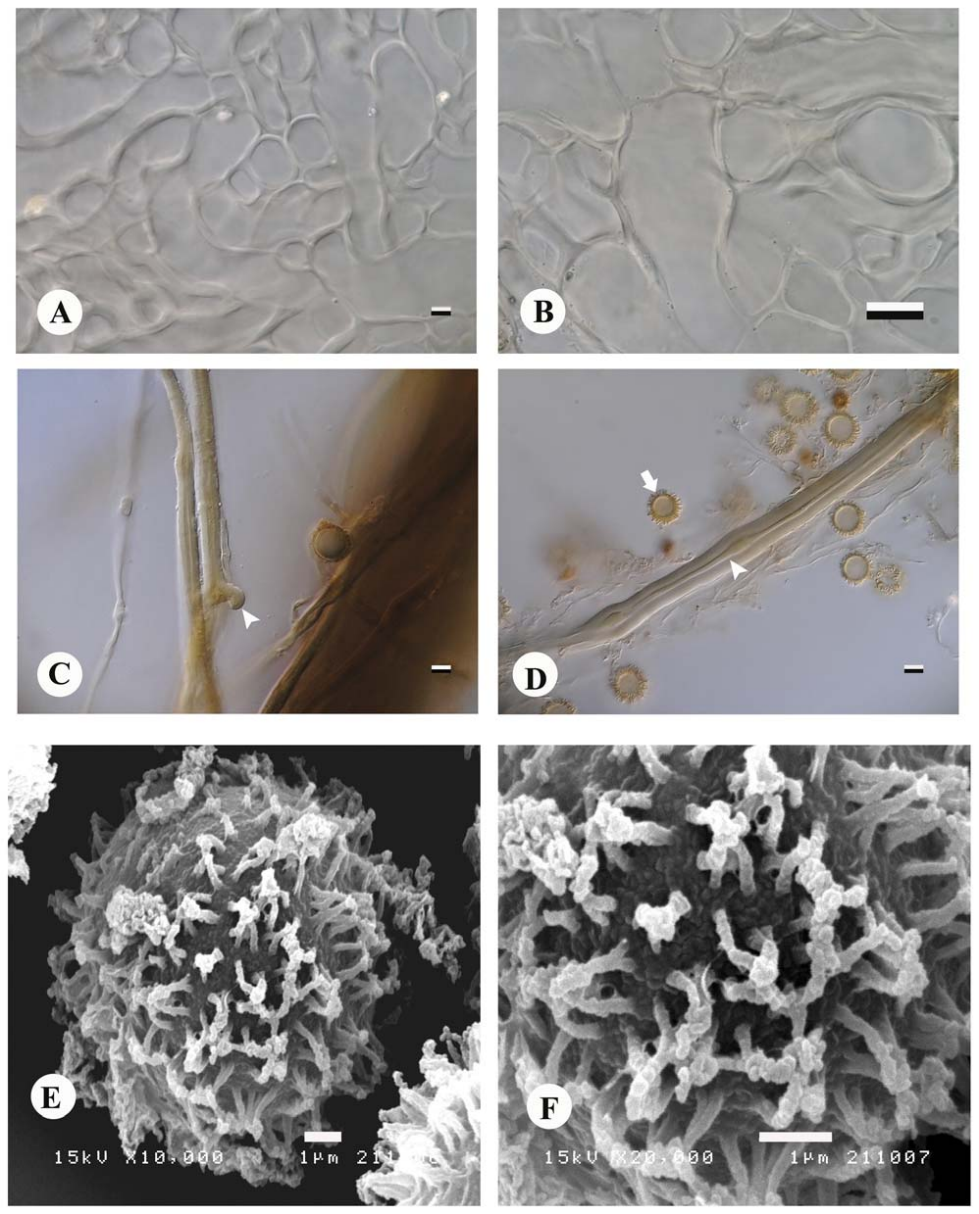
*Astraeus sirindhorniae*. Exoperidium layers (A–B). (A) exoperidial subpellis, bar = 5 µm. (B) exoperidial subpellis (innermost), bar = 10 µm. (C) rhizomorph hyphae with clamp connection (arrowhead), bar = 5 µm. (D) capillitium hyphae displaying continuous lumen (arrowhead) and basidiospore (arrow), bar = 5 µm. (E–F) spore ornamentation demonstrated coalescent spines in groups, bar = 1 µm. A–D magnification at 1,000×.

Basidiomes subglobose to ellipsoid at first (Fig. 4A), slightly compressed, hard, subepigeous 24.5–55.0 mm diam., dry, with woolly, adpressed covering forming felty, adpressed triangular to hexagonal scales, denser and more fluffy towards base where they are intermixed with date-brown to sepia rhizomorphs (Fig. 1A), splitting into concentric zones which fuse towards uppermost, exoposed parts, with thick, complex exoperidium (Fig. 4E), expanding to become star-shaped and then 40–100 mm broad, tough, surface often encrusted with soil particles; odour strong, penetrating, pleasant. When mature exoperidium buff to snuff-brown, squamulose, consisting of at least 3 distinct layers 3–5 mm thick when fresh, contracting to <1 mm when dry, leathery, splitting into 6–8 broad, stellate rays, innermost layer varying from buff to brownish, extensively scaly cracked to give almost regular pattern. Endoperidium shortly stipitate (Fig. 4B), globose to subglobose ca 18–32 µm diam., white at first, becoming buff to hazel when mature, very fluffy-fibrillose (Fig. 4C), even velvety opening by apical, irregular tear and lacking defined peristome. Gleba purplish chestnut becoming umber to date-brown when mature (Fig. 4D), lacking columella. Exoperidial suprapellis ca 70–80 µm, brownish, consisting of interwoven, periclinal to perpendicular, thin- or thick-walled hyphae 4–7 µm broad, with central lumen and walls 1–2 µm thick (Fig. 5A–C). Exoperidial mediopellis, fibrous ca 600 µm broad of interwoven periclinal to orthogonal hyphae 5–7 µm broad with hyaline, continuous lumen and walls 1–2 µm thick and becoming more parallel at junction with subpellis (Fig. 5C–F). Exoperidial subpellis pseudoparachymatous, ca 1050–1100 µm broad of hyaline, parallel to anticlinal, thick-walled hyphae with walls 1–2 µm thick (Fig. 5F and Fig. 6A–B). Clamp-connections absent in exoperidium. Endoperidium consisting of brownish, interwoven, unbranched, aseptate hyphae ca 5–8 µm broad with continous lumen and walls 1–2 µm thick, clamp-connections absent. Capillitium of long, unbranched, interwoven, hyaline, aseptate threads 4–7 µm broad and lacking clamp-connections (Fig. 6D). Basidiospores globose, (5.19)-6–11 µm diam., including ornamentation, umber to date-brown (Fig. 6D), with moderately dense, rounded, narrow, tapered, separate tubercles which coalesce in groups (Fig. 6 E–F).

#### Habitat

In rainy season, gregarious, partially buried in ultisols in dry deciduous forests associated with *Dipterocarpus tuberculatus* Roxb., *Shorea obtusa* Wall. and *Shorea siamensis* Miq.

#### Distribution

North and Northeastern areas of Thailand.

#### Material examined

Thailand, Chiyaphum province, Phu khieo Wildlife Sanctuary, Dipterocarp forests, N 16°27′32″ and E 101°39′414″, elev. 640 msl, 9 September 2010 (BBH 34830, duplicate E30288, duplicate MA-Fungi 82080); Ibidem, date, (BBH 34831), Mae Cham district, Dipterocarp forests, N 18°31′981″ and E 98°24′939″, June–September 2010.

#### Note

Her Royal Highness the Crown Princess of Thailand, has considered and granted for a new fungus name; *A. sirindhornii*. This name is a great honor and a privilege. However according to ICBN Recommendation 60C.1(b) If the personal name ends with a consonant (but not in *-er*), substantival epithets are formed by adding *-i-* (stem augmentation) plus the genitive inflection appropriate to the sex and number of the person(s) honoured (e.g. *lecard-ii* for Lecard (m), *wilson-iae* for Wilson (f), *verlot-iorum* for the Verlot brothers, *braun-iarum* for the Braun sisters, *mason-iorum* for Mason, father and daughter). Therefore *A. sirindhornii* should ending with –iae and then the epithet to be spelled; *A. sirindhorniae*.

## Discussion

*Astraeus sirindhorniae* represents a new species of star-shaped gasteroid fungus which differs morphologically from many other genera of star-shaped fungi. In comparing this species, the earthstar genus, *Geastrum*, tends to have a well defined peristome. *Myriostoma* species may be distinguished by the formation of multiple irregular shaped peristomes from which spores escape. *Trichaster* differs in having an endoperidium that remains attached to the exoperidium after opening, then soon disintegrates leaving a powdery spore-mass suppported by a stout, persistent collumella. The endoperidium of *Terrostella* is thin and peels away to expose a powdery spore-mass supported by a distinct sterile base. *Phialastrum* has a strongly developed columella and *Geasteropsis* produces an extremely hard basidiome when dry.

According to Phosri et al. there are only two *Astraeus* species in Thailand, *A. odoratus* and *A. asiaticus* [23], [24]. *Astraeus odoratus* is found under ecological conditions similar to those at the Phu Khieo Wildlife Sanctuary. However, *A. sirindhorniae* differs in its much larger basidiomes, both when immature and when its rays are fully expanded, displaying flared margins, and exposing complex layering. *Astraeus sirindhorniae* is further differentiated from *A. odoratus* through the presence of prominent rhizomorphs, a complex multi-layered exoperidium, and smaller basidiospores (range 6–11 µm). These basidiospores are also smaller than *A. asiaticus* spores (8.75–15.2 µm) and generally given for *A. hygrometricus s. str. viz*. (7.5–12 µm) [25], [26], [27], [28], [29]. The spore ornamentation of *A. sirindhorniae* is notable under SEM as it has moderately dense, rounded, narrow, tapered, separate tubercles, which coalesce spines in groups. In addition, *A. sirindhorniae* has a short stipitate, very fluffly- fibrillose endoperidium when immature, which further differentiates this taxon from other *Astraeus* species.

The outermost felty, scaly covering of the young basidiomes of *A. sirindhorniae* closely resembles that of members of *Scleroderma* previously placed in *Veligaster*. The gleba is probably not divided into tramal plates. As in *A. sirindhorniae* clamp-connections are absent from both the gleba and the peridium. On maturing the highly gelatinized middle layer is exposed well before the powdery gleba is revealed. In *A. sirindhorniae* the peridial medio- and subpeillis are not gelatinized and the hyphae are fully differentiated but otherwise the very young basidiomes are similar in primordial structure.

In the multi-gene phylogenetic analyses *A. sirindhorniae*, along with other *Astraeus* species, form a monophyletic clade with *Tremellogaster*, and *Diplocystis* (Fig. 2). This clade is recognized as the Diplocystidiaceae. The structure of the peridium in *Tremellogaster* is also rather complex: the outer wall consists of thickened, sclerotised hyphae; the middle layer is brown and heavily gelatinised and divided into polygonal areas of plate-like, non-gelatinous tissue; and the innermost layer consisting of hyaline, thin-walled hyphae similar to those in *A. sirindhorniae* but posses transverse thickenings. A summary of the pertinent characters and literature references for *Tremellogaster* are given in Watling [30].

Members of the Sclerodermatineae form ectomycorrhizal associations with many host plants. Species of *Astraeus* are known to associate with ectomycorrhizal plant hosts in the Pinaceae, Betulaceae, Fagaceae, Ericaceae and Dipterocarpaceae [31]. Given its phylogenetic placement, and the fact that it is found in dipterocarp dominated forests, it is likely that *A. sirindhorniae* is also an ectomycorrhizal fungus. Further study into the ecology of this species is needed in order to conclusively identify possible relationships to dipterocarpacious hosts.

In the multigene phylogeny the basal position of *A. sirindhorniae* relative to other *Astraeus* taxa is interesting from a biogeographic standpoint (Fig. 2). This placement suggests a Southeast Asian origin for the genus, which is observed in many Sclerodermatineae genera [31]. However, this is complicated by the fact that the basal Diplocystidiaceae (*Diplocystis* and *Tremellogaster*) are monotypic genera whose species are described from the new world (the Caribbean and South America respectively). Further investigation into the biogeogaphic history of these taxa is necessary to understand the current distribution of new- and old-world *Astraeus*.

## Conclusions

In summary *A. sirindhorniae* is morphologically distinguished from *A. odoratus*, *A. asiaticus* and *A. hygrometricus s.l.* by basidiome and basidiospore size, spore ornamentation and peridium structure. Phylogenetic analysis clearly resolves *Astraeus sirindhorniae* as a basal lineage of *Astraeus*, within the Diplocystidiaceae and Sclerodermatineae. This systematic relationship, in combination with its associations with dipterocarp forests, it is probable that this species is ectomycorrhizal with members of the Dipterocarpaceae. *Astraeus sirindhorniae* represents a new gasteroid, star-shaped fungus from Thailand. This discovery reinforces the belief that fungi represent a group of organisms with many undescribed taxa; some of which exist within the dry evergreen dipterocarp forests of SE Asia.
